# Supplementary material for: Quantitative, high-sensitivity measurement of liquid analytes using a smartphone compass
Source: Nat Commun. 2024 Mar 30;15:2801. doi: 10.1038/s41467-024-47073-2 (PMC10981709; doi:10.1038/s41467-024-47073-2)
Supplement: Supplementary file 1 — Supplementary Information [file 41467_2024_47073_MOESM1_ESM.pdf]

Supplementary Information for

Quantitative, high sensitivity measurement of liquid analytes using a smartphone compass

Mark Ferris[1,2], Gary Zabow[1,*]

[1]Applied Physics Division, National Institute of Standards and Technology, Boulder, Colorado, 80305

[2]Department of Physics, University of Colorado, Boulder, Colorado, 80309

*Gary Zabow

Email: gary.zabow@nist.gov

Table of Contents

1. Supplementary Notes: magnetometer-based smartphone sensor platform

    1. Supplementary Figure 1: Magnetometer-based smartphone sensor used for data collection on Motorola Moto E (2020)

    2. Supplementary Figure 2: Blueprints for Motorola Moto E attachment piece

    3. Supplementary Figure 3: Attachment piece designed for Google Pixel 2

    4. Supplementary Figure 4: Blueprints for Google Pixel 2 attachment piece

    5. Supplementary Figure 5: Control of glucose bilayer curvature with DMA.

2. Supplementary Notes: system characterization

    1. Supplementary Figure 6: Determination of the magnetometer-based smartphone sensor signal

    2. Supplementary Figure 7: Full Reversibility data for glucose cycle 1

    3. Supplementary Figure 8: Magnetometer-based glucose platform signal for the full reversibility test

    4. Supplementary Figure 9: Response time of the glucose hydrogel actuator to 20 mM glucose

    5. Supplementary Figure 10: Reversibility of the pH hydrogel actuator between pH 4 and pH 7 buffers

    6. Supplementary Figure 11: Reproducibility of response for the pH sensor platform and phone model comparison

    7. Supplementary Figure 12: Summary of pH hydrogel reproducibility

    8. Supplementary Figure 13: pH dose/response calibration supporting data

    9. Supplementary Figure 14: Improving the hydrogel actuator response time

3. Supplementary Notes: detection limit results

    1. Supplementary Figure 15: Supplementary data for detection limit testing in Fig. 4a

    2. Supplementary Figure 16: Supplementary data for the noise analysis in Fig. 4b

    3. Supplementary Figure 17: Evaluation of noise from the dose/response data in Fig. 2b

4. Supplementary Discussion

    1. Magnetic field as a function of bilayer curvature

        1. Supplementary Figure 18: Bilayer geometry

        2. Supplementary Figure 19: Bilayer curves

    2. Range of validity of on-axis disk field model (main paper Equation 1)

        1. Supplementary Figure 20: Field change ΔB_z as a function of hydrogel fractional expansion ε

    3. Comparison of bilayer vs monolayer

        1. Supplementary Figure 21: Comparison of bilayer versus monolayer sensitivity

5. Supplementary Tables: Cost estimates

    1. Supplementary Table 1: Cost estimate for glucose Hydrogel Actuator

    2. Supplementary Table 2: Cost estimate for pH Hydrogel Actuator

6. Supplementary Methods

1. Supplementary Notes: magnetometer-based smartphone sensor platform

For data collection and initial testing and development, it was beneficial to be able to quickly adjust the fit of the attachment piece with shims so that the magnetic particles could be moved directly over the magnetometer for various iterations of the hydrogel actuator during development. We used many differently shaped attachment pieces including those shown in Supplementary Figs. 1a and 1b. The prototype platform suggested in main paper Figs. 1a and 1b represents a simplified, more compact version we envision for future use, where the attachment piece snaps snugly onto the phone without shims and with the magnetic particles of the hydrogel actuator automatically positioned directly over the magnetometer. The size and formulation of the hydrogel actuator would need to be standardized to use the type of assembly shown in the prototype.

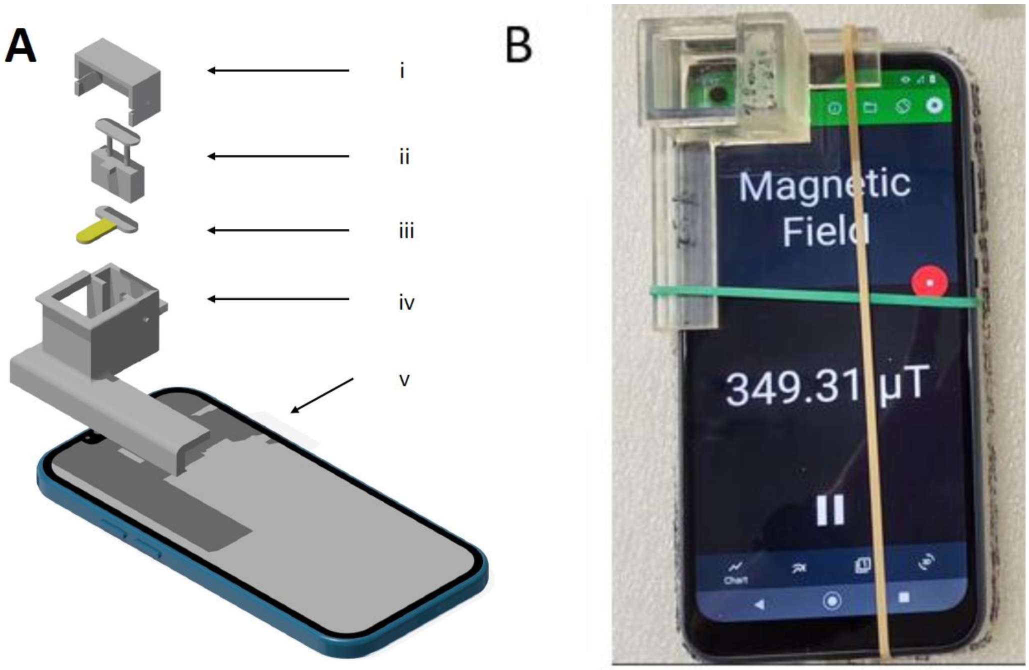

Supplementary Figure 1: Magnetic hydrogel smartphone sensor platform used for data collection in this paper. *(a)* Schematic of the full assembly, including (i) lid, (ii) positioning clamp, (iii) hydrogel actuator, (iv) attachment piece with analyte well, and (v) smartphone. Shims are placed in between the attachment piece and the smartphone on the top and left sides to position the magnetic particles directly over the smartphone magnetometer. *(b)* Photograph of the assembled magnetometer-based smartphone sensor.

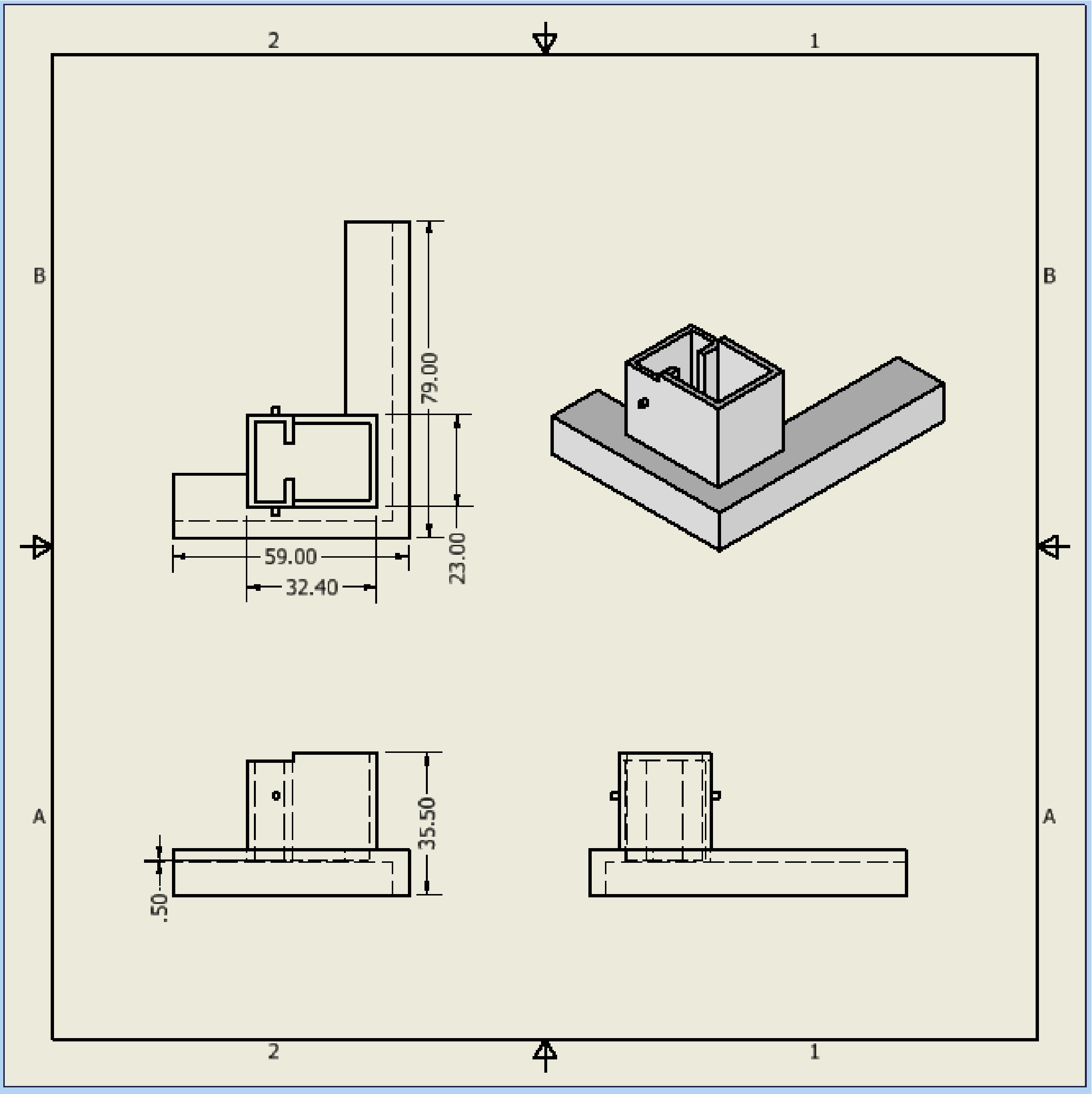

Supplementary Figure 2: Blueprints for the Motorola Moto E (2020) attachment piece. Labelled dimensions are in millimeters. Selected dimensions are labelled to give a sense for the overall size of the attachment piece.

Adapting the magnetometer-based smartphone sensor to other smartphone models requires only changing the location of the analyte well to place the magnetic material over the magnetometer of the smartphone. As an example of a different phone, Supplementary Fig. 3 shows an attachment piece designed for a Google Pixel 2 smartphone.

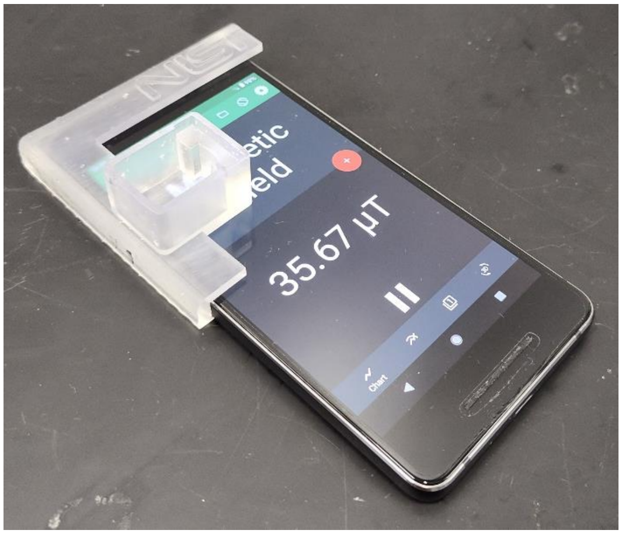

Supplementary Figure 3: Picture of a version of the plastic attachment piece designed for the Google Pixel 2.

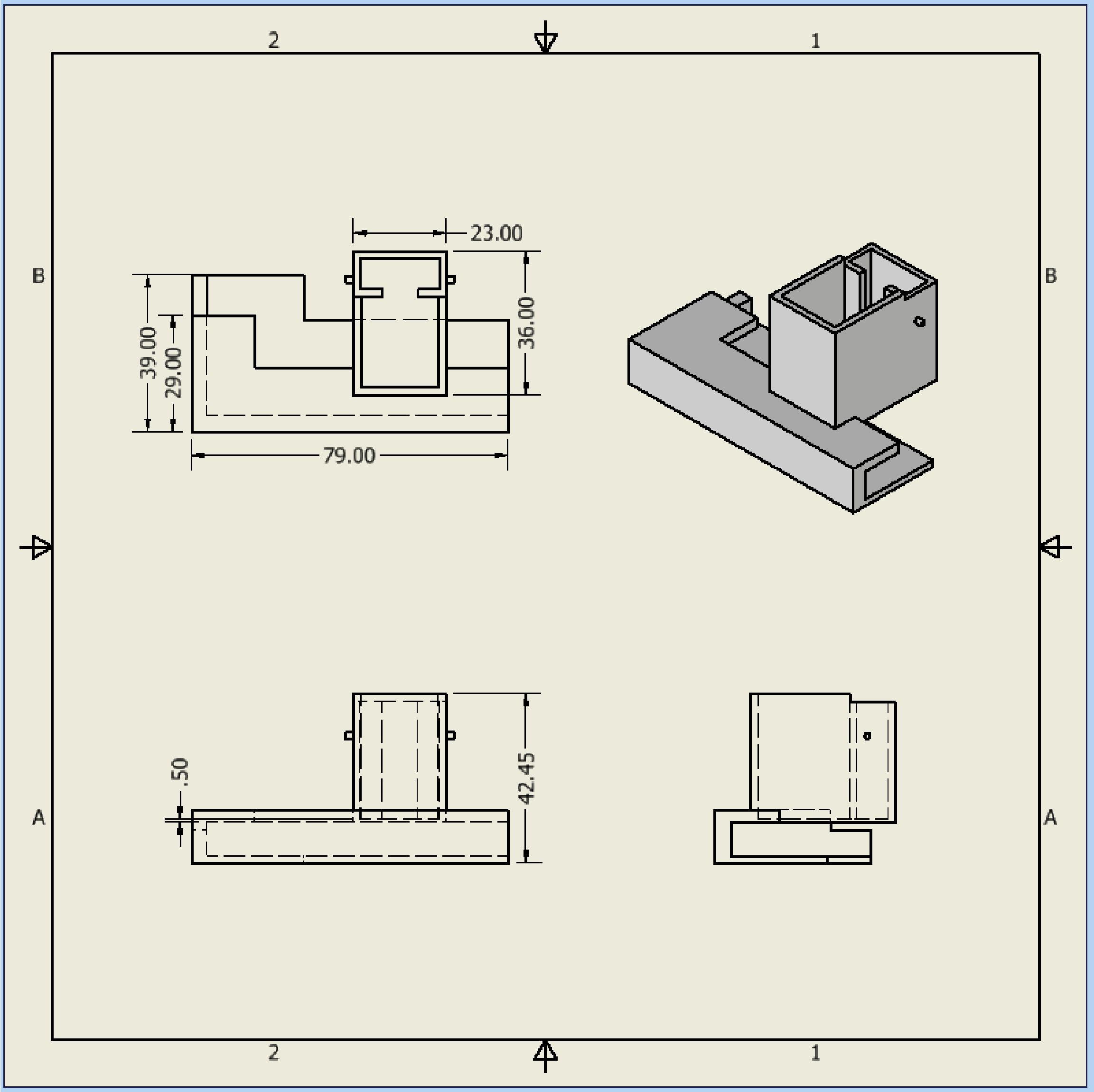

Supplementary Figure 4: Blueprints for a version of the attachment piece designed to fit the Google Pixel 2.

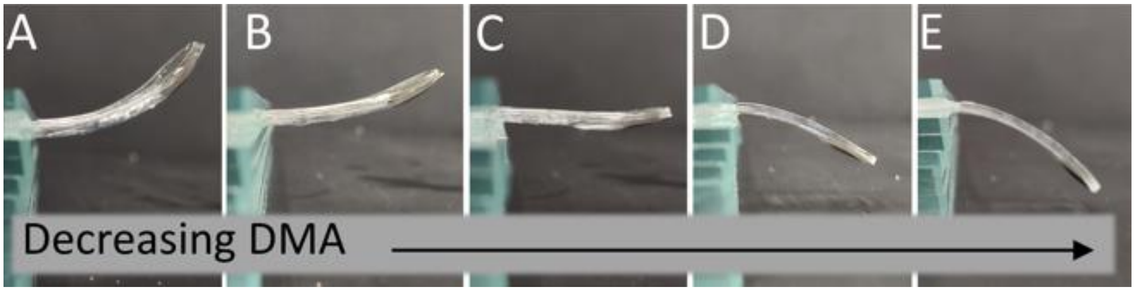

Supplementary Figure 5: Control of the initial, analyte-free curvatures of the glucose hydrogel actuators after swelling to equilibrium in pH = 7.4 buffer with 0 mM glucose by using inert layers containing *(a)* 80 μL, *(b)* 70 μL, *(c)* 60 μL, (*d*) 50 μL, and (*e*) 40 μL of DMA. Varying the content of DMA in the inert layer compared to the smart hydrogel layer allows the bilayer to lay flat for any chosen initial analyte concentration.

2. Supplementary Notes: system characterization

To ascertain whether potential fluctuations in the background magnetic field might interfere with the sensor readings, two smartphones were used for data collection, one containing the full sensor platform (sensor phone) and one stationed nearby with no additional hardware (background phone). Both phones were calibrated to the background magnetic field before testing. Supplementary Figure 6 shows a sample of the raw data collected (from Fig. 2B, 20 mM reversibility cycle 2) during an experiment. The magnetic field reading, *B*, from the background phone (Supplementary Figure 6a) confirms that there is little change in the background magnetic field over the course of an experiment, with total fluctuations in the vector components B_x, B_y, B_z, and in B_t (the total field) each on the order of ~1 μT or less. The magnetic field reading, *B,* of the sensor phone (Supplementary Figure 6b) shows that most of the change in magnetic field in response to 20 mM glucose is a result of sensor actuation is in the z-direction, with minimal changes in B_x and B_y. The change in B_t closely mirrors the change in B_z, which has a large negative value. This is expected since the embedded ensemble of $Nd_2Fe_{14}B$ particles were magnetized axially and with negative z-direction pointed downward into the phone. The particles were magnetized axially instead of in-plane to maximize the magnetic field reading. The magnetic field reading of the background phone was subtracted from that of the sensor phone to obtain a background-subtracted signal where variations in the background magnetic field are controlled for (Supplementary Figure 6c). The signal of the magnetometer-based smartphone glucose sensor is generally considered to be the change in B_t from a 0 mM glucose baseline of the background-subtracted signal (Supplementary Figure 6d). In this work, B_x and B_y are negligible, making B_t approximately equal to B_z, and so from here on we refer to the signal of the magnetometer-based smartphone glucose sensor as ΔB_z. While full background subtracted data is presented here for completeness, it should be noted that such background subtraction is only necessary in noisy magnetic environments. In practice there was never a substantial difference between the raw (sensor phone) and background-subtracted data in any of the data reported in this report, and therefore only one phone is necessary for implementation of the magnetometer-based smartphone sensor.

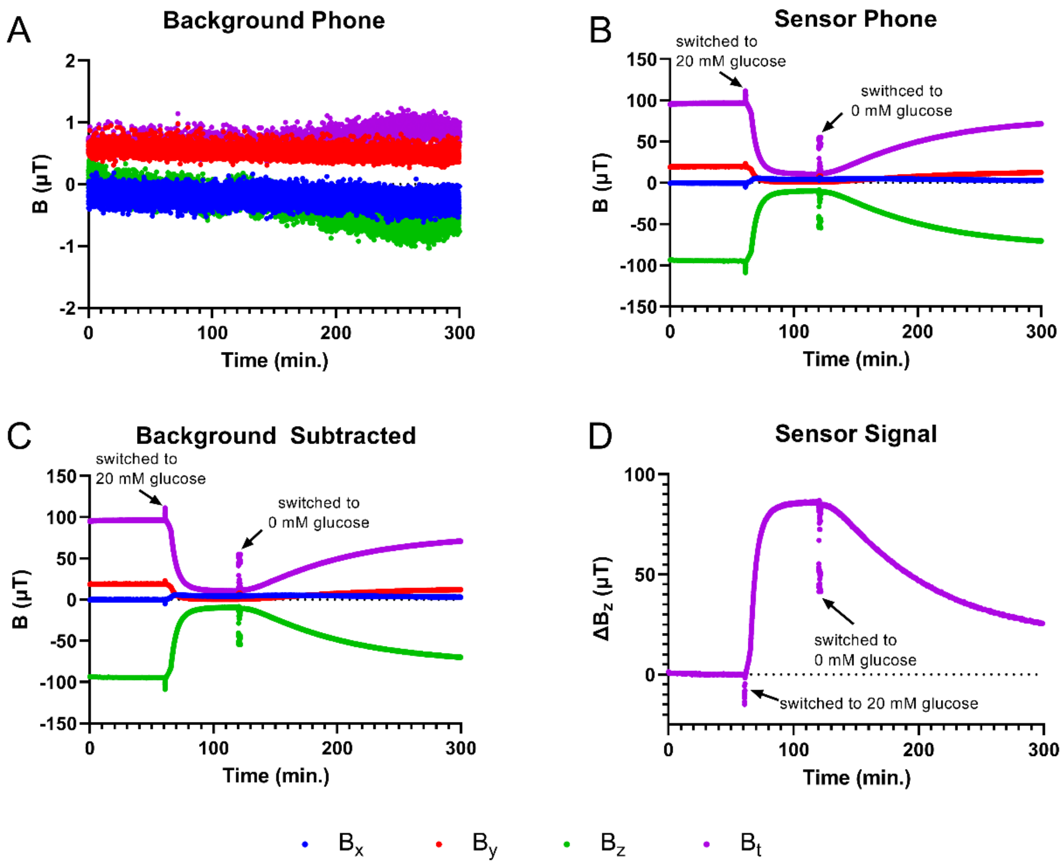

Supplementary Figure 6: Determination of the magnetometer-based smartphone sensor signal. Raw magnetometer readings (*b*) collected from 3-axis magnetometer in the *(a)* background phone, *(b)* test phone, and *(c)* the resulting background-subtracted sensor signal. *(d)* The signal of the magnetometer-based smartphone glucose sensor is generally considered to be the change in field strength of the background-subtracted total field strength (ΔB_z) from the 0 mM glucose baseline. Source data are provided as a Source Data file.

The reverse response of the hydrogel actuator (going from high glucose concentration to low glucose concentration) is slower than the forward response (0 to 20 mM). For each cycle, the magnetic field reading (B*)* was recorded in 0 mM glucose solution for 1 hour, changed to 20 mM glucose for 1 hour, then changed back to 0 mM glucose. The 0 mM glucose solution is then refreshed several times over several hours to return the magnetometer reading to its initial value (Supplementary Figure 7). Supplementary Figure 8 shows the ΔB_z over three cycles of activation with 20 mM glucose. Solution changes are marked by brief downward spikes in the trendline, which occur due to the hydrogel actuator being pressed flat against the bottom of the well due to capillary forces in the absence of solution. Due to the high responsiveness of the sensor at low concentrations of glucose, thorough washing is required to return the magnetometer reading fully to baseline. Slow elastic relaxation of the hydrogel may also contribute to a slower reversible response of the sensor, even after all remnant glucose has been washed out, such as can be seen after the final wash step in cycle 3 (Supplementary Fig. 8c). While the low cost of the device allows for its use as a disposable test strip, this data shows that with further development (i.e. increased response time), the sensor has the potential for continuous monitoring applications.

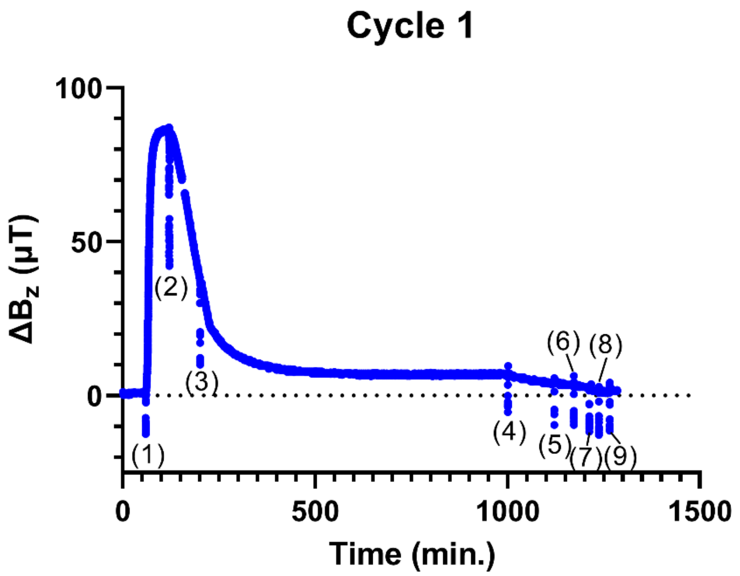

Supplementary Figure 7: Full Reversibility data for glucose cycle 1. Several wash cycles are required to fully return the magnetometer-based glucose sensor signal to baseline. The sensor begins in a 0 mM glucose solution which is switched to 20 mM at time point (1). The solution is returned to 0 mM at time point (2) and this 0 mM solution is refreshed at timepoints (3) through (9) to account for any residual glucose that might not have been fully washed out during the prior steps. Source data are provided as a Source Data file.

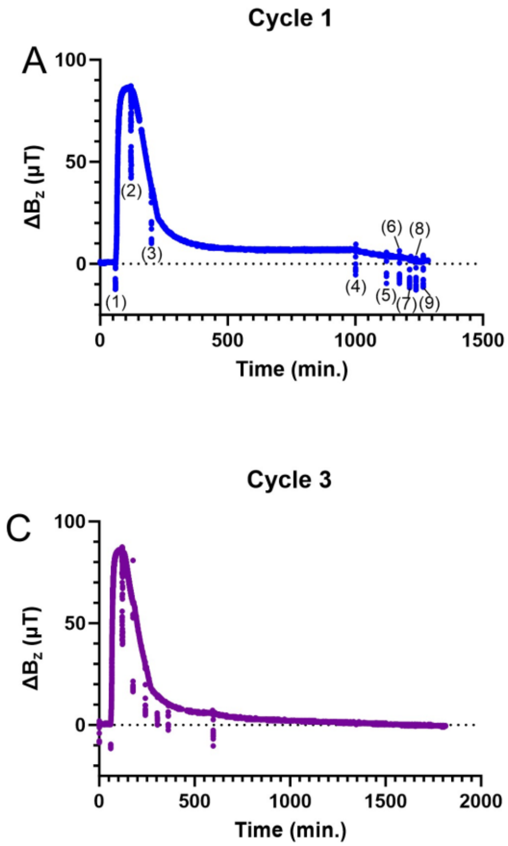

Supplementary Figure 8: Magnetometer-based glucose platform signal for the full reversibility test in *(a)* Cycle 1, *(b)* Cycle 2, *(c)* Cycle 3. For each cycle, the sensor reading was recorded in 0 mM glucose solution for 1 hour, changed to 20 mM glucose for 1 hour, then changed back to 0 mM glucose. The 0 mM glucose solution must be refreshed several times to return the magnetometer reading to its initial value. Differences in relaxation behavior back to baseline may be due to differences in when, and how thoroughly, wash steps were performed as well as due to potential diffusive mixing of possible remnant glucose in the test well, but once the sensor is back to baseline, it responds again reproducibly to the addition of glucose. Source data are provided as a Source Data file.

Supplementary Figure 9 shows the change in field strength (*ΔB*$_z$) in response to the three cycles of 20 mM glucose fit to a one-phase association curve,

$$Y = Y_0 + (Plateau - Y_0) * (1 - exp(-K * x))$$

with GraphPad Prism Software version 9.4.0, Y_0 is the y-intercept, *Plateau* is final value of the response, and *k* is a rate constant. Data before the inflection point in the response, seen at ~4.5 minutes in all three cycles, were excluded from the fit. This initial phase of the response is explained by the hydrogel resting on a bar placed toward the end of the bilayer section of the actuator while the bilayer is mostly flat in 0 mM conditions. This supporting bar was only necessary to prevent an initial response where the magnetometer reading decreased instead of increased, which occurred only in iterations of test hydrogel actuators where the initial curvature of the bilayer was slightly downward instead of flat or slightly upward. The slower initial phase of the response corresponds to the bilayer changing its curvature from downwards to upwards while being supported by the bar, and the accelerated rate of response after the inflection point at ~4.5 minutes corresponds to when the sensor has activated sufficiently such that it is no longer supported by the bar and is now freely travelling upwards and away from the phone surface through the 20 mM glucose test solution. The slope increases roughly 4x at this point, meaning a factor of four sensitivity is likely lost at the low end of the response, before the hydrogel actuator lifts off the bottom of the well. If the initial curvature of the bilayer is flat or slightly upwards, such as the iteration used in the future detection limit test, then the bar is not necessary (which should be the case for all future systems). Shims can also be used underneath the inert, horizontal section of the hydrogel actuator to raise it up so that the bilayer section does not rest on the bottom. In these cases, the response is single phase. The response time (T_{90}) was defined as the time it takes for ΔB_z to reach 90% of the plateau value. The T_{90} of the measured response to 20 mM glucose was determined to be 19.4, 19.0, and 19.6 minutes for cycles 1, 2, and 3, respectively.

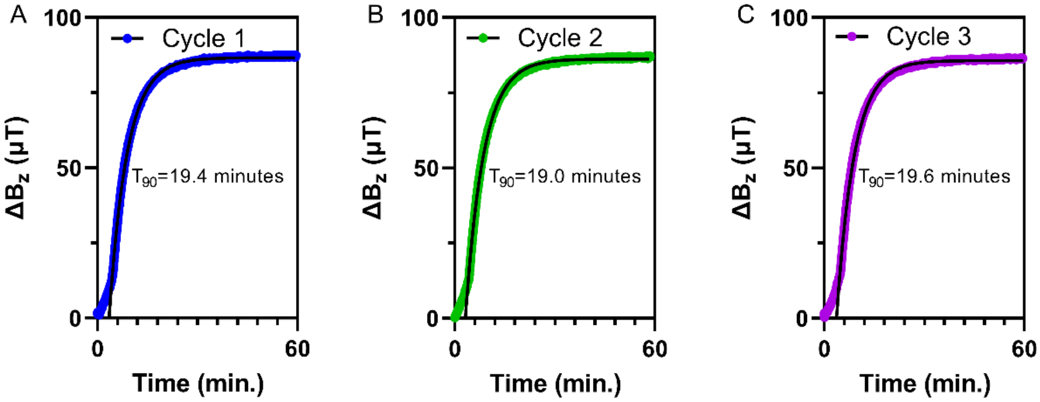

Supplementary Figure 9: Response time of the same glucose hydrogel actuator to 20 mM glucose for *(a)* Cycle 1, *(b)* Cycle 2, and *(c)* Cycle 3. Source data are provided as a Source Data file.

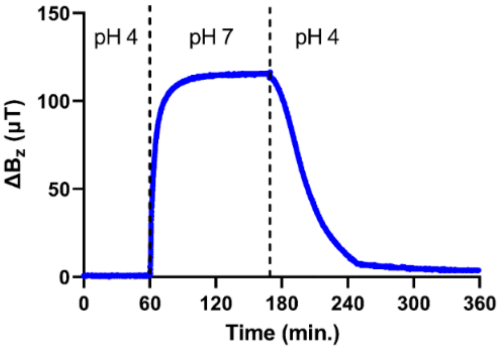

Supplementary Figure 10: Response of the pH hydrogel actuator between pH 4 and pH 7 buffers.

The pH-responsive platform was characterized in terms of reversibility (Supplementary Fig. 10), reproducibility of the response (Supplementary Figs. 11 and 12), and dose/response calibration (Fig. 3b in the main paper with supporting data in Supplementary Fig. 13). The pH hydrogel actuator is designed to lay flat in pH 4 solution. When the buffer is changed to pH 7, the bilayer portion of the hydrogel actuator curls and moves the $Nd_2Fe_{14}B$ away from the magnetometer, increasing ΔB_z. When the solution is changed back to pH 4, the bilayer flattens and the magnetometer reading returns to baseline, as shown in Supplementary Fig. 10. Here, the response of a single pH hydrogel actuator from pH 4 to 5 was tested 3x each on the Motorola Moto E (2020) and Google Pixel 2, alternating between the two, with the resulting response curves shown individually in Supplementary Figs. 11a – S11f. Then, the response was again tested but with the Google Pixel 2 attachment piece affixed to the Moto E,but appropriately laterally shifted for different magnetometer placement in such a way that the magnetic field reading was maximized when the bilayer was flat. The hydrogel actuator was reset to baseline in pH 4 buffer before each trial, and shims were used underneath the inert clamping region of the actuator to adjust its position vertically, so that the baseline (pH 4) magnetometer value matched for each trial. A noisier signal is seen in the Google Pixel 2 trials, indicating that this (refurbished) phone model contained a noisier magnetometer than the one in the Moto E.

Supplementary Figure 12a shows the three response curves from the Moto E trials overlaid. The response is highly repeatable, with the standard deviation of the endpoint magnetometer response being just 0.56% of the total change in magnetic field reading. Supplementary Figure 12b shows the three response curves from the Google Pixel 2 trials overlaid. The response is also highly repeatable on this phone model, with the standard deviation of the endpoint magnetometer response being just 1.2% of the total change in magnetic field reading. The response is also reproducible between the phone models. Supplementary Figure 12c shows the 3rd magnetometer response curve from both the Moto E and the Google Pixel 2, as well as the control trial where the Google Pixel 2 attachment piece was affixed to the Motorola Moto E. This plot shows that the shape of the response curve is only slightly different when using the Moto E vs. the Google Pixel 2. The control trial (yellow) matches the standard Motorola Moto E trial (orange), indicating that the difference in the shape of the response curves is due to a difference in the sensitivities of the two magnetometers at different points in space, rather than being an artifact of the different attachment pieces. Despite the difference in the shape of the response curves, the endpoint magnetometer readings are in good agreement. Supplementary Figure 12d shows the average and standard deviation of the endpoint magnetometer response for the three Moto E trials and the three Google Pixel 2 trials, with a difference of just 2.7 μT. This data shows that the response of the pH platform is also stable over at least 4 days (the duration over which these multiple experiments were performed) and many actuation and washing cycles.

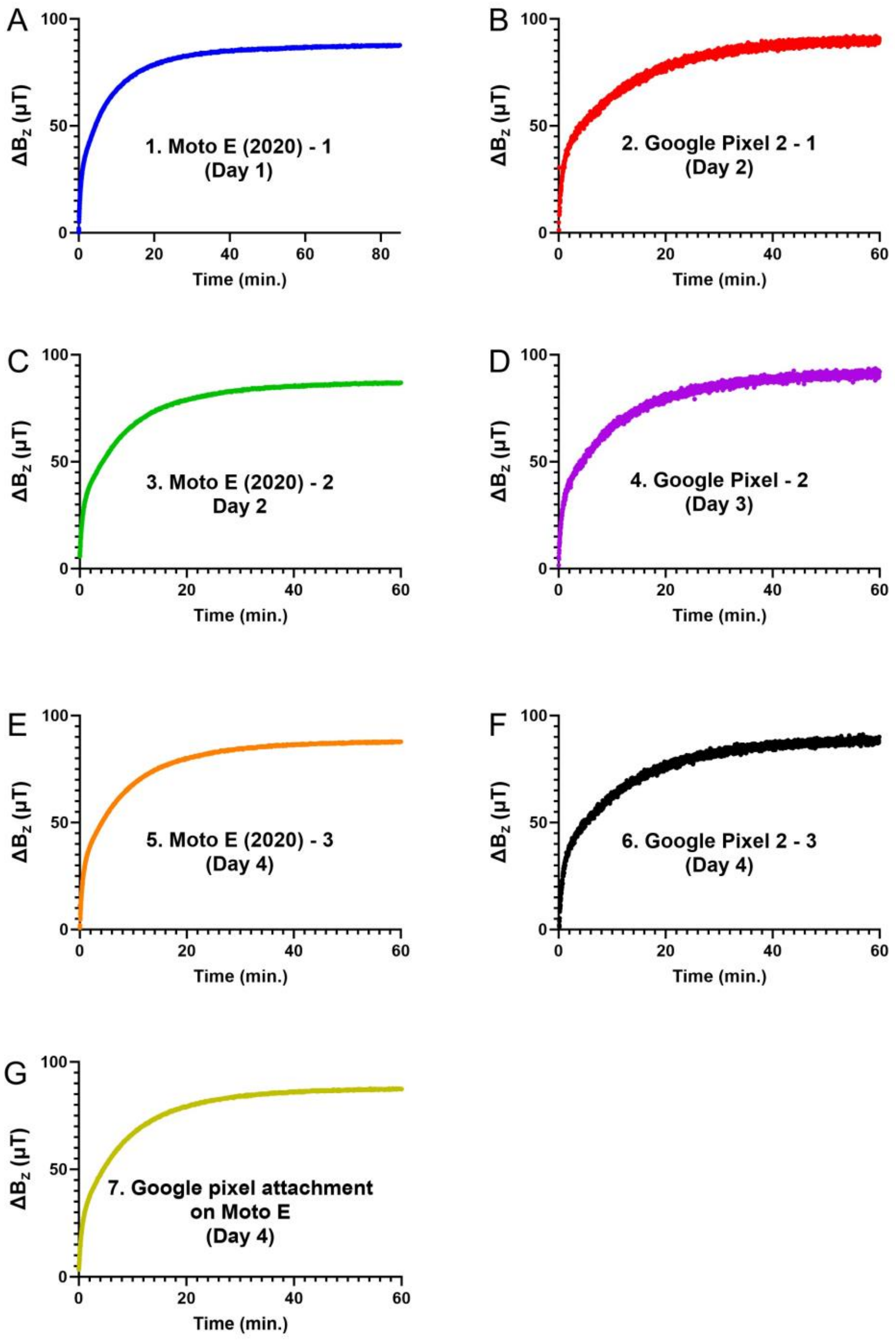

Supplementary Figure 11: Reproducibility of response for the pH sensor platform and phone model comparison. The response (*ΔB_z*) of a single pH hydrogel actuator, from pH 4 to pH 5 buffer, alternating between testing on *(a, c, e)* the Motorola Moto E (2020) and *(b, d, f)* the Google Pixel 2 over 4 days. *(g)* The response (*ΔB_z*) of the same pH hydrogel actuator, from a pH 4 buffer to a pH 5 buffer, on the Moto E but using the Google Pixel 2 attachment piece. Source data are provided as a Source Data file.

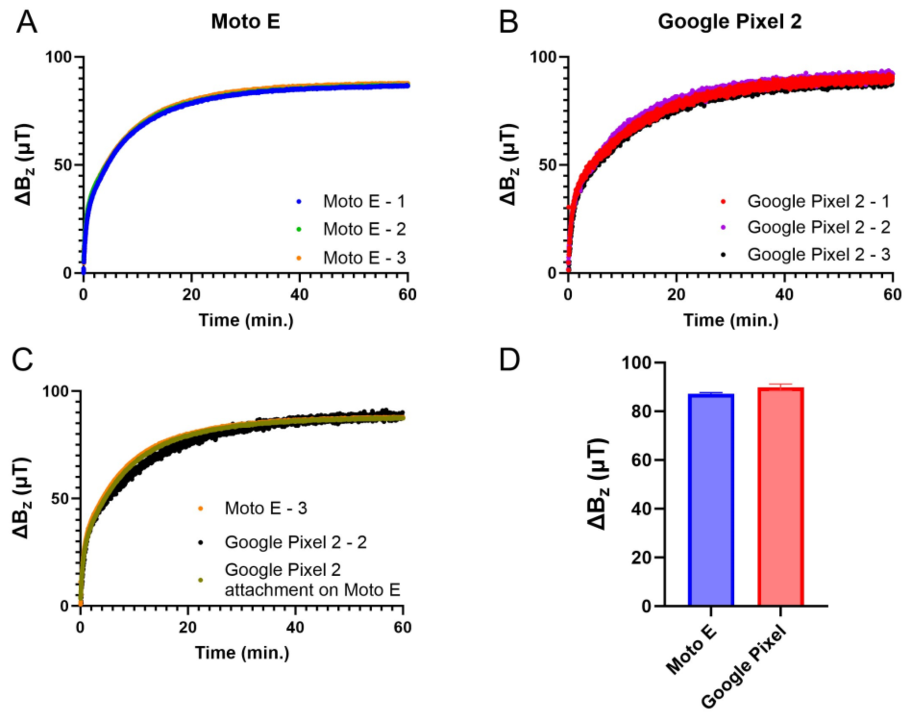

Supplementary Figure 12: Summary of pH hydrogel reproducibility. *(a)* Response of the pH-responsive hydrogel actuator on a Motorola Moto E from pH 4 to pH 5 buffer for three trials (data shown individually in Supplementary Figs. 11 a, c, and e). *(b)* Response of the pH-responsive hydrogel actuator on a Google Pixel 2 from pH 4 buffer to pH 5 buffer for three trials (data shown individually in Supplementary Figs. 11 b, d, and f). *(c)* Comparison of the 3rd Moto E trial, the 3rd Google Pixel 2 trial, and the Moto E response using the Google Pixel 2 attachment piece, showing that the response curve of the Moto E using the Google Pixel 2 attachment matches the response curve of the other Moto E trials rather than the other Google Pixel 2 trials. *(d)* Average endpoint magnetometer response from pH 4 to pH 5 buffer on the Moto E, vs the Google Pixel 2. Source data are provided as a Source Data file.

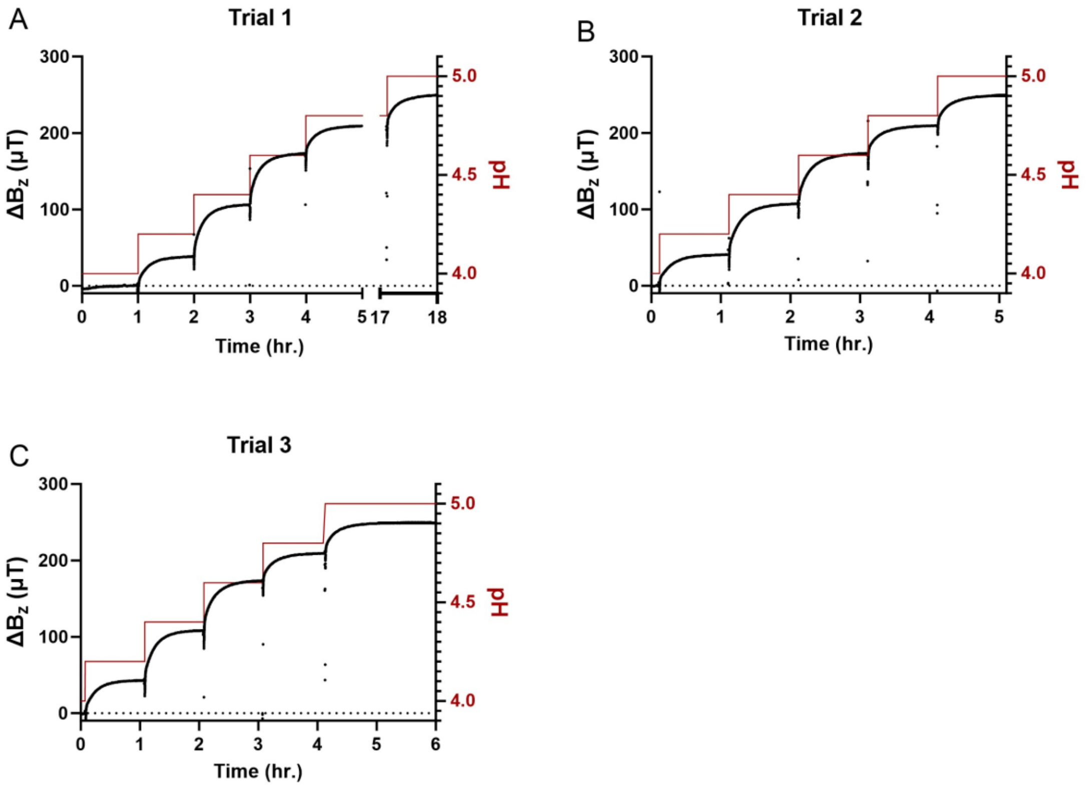

Supplementary Figure 13: pH dose/response calibration supporting data for Fig. 3b in the main paper. Plots show *ΔB_z* over time, with the pH test solution incremented by 0.2 pH units periodically, starting at pH 4 and ending with pH 5 for *(a)* trial 1, *(b)* trial 2, and *(c)* trial 3. Source data are provided as a Source Data file.

Supplementary Figure 14 shows that the response time of a hydrogel actuator is improved by adding Polyethylene Glycol 10,000 (PEG), serving as a pore-forming agent, to the precursor solutions for both the inert and active layers, as well as by making the active layer thinner. This was demonstrated on a pH-responsive hydrogel but could be applied to the glucose hydrogel actuator as well. Supplementary Figure 14a shows the raw field strength recording for pH hydrogels made with (red) and without (blue) PEG, and with both PEG and a thinner active layer (green), and the resulting response curve when the test solution is changed from pH 4 to pH 5, causing the actuators to curl from an initial flat state. The background-subtracted field strength is plotted rather than ΔB_z to show that the response curves plateau at or above ~26 μT, meaning that they are still well within range of the magnetometer. This was done to avoid response times of the hydrogel actuators that may appear artificially fast if the bilayers curl to a degree where the magnetic particles move out of range and the magnetometer reads ~0 μT. The red, blue, and green curves start at different field strengths only because the different actuators had different initial offset distances from the base of the test fluid container and/or different magnetic particle loadings; this does not affect response time as can be seen in Supplementary Fig. 14b which shows the normalized response curves, clearly demonstrating the improved response time when PEG is added and/or the bilayer is made thinner. The T_{90} of these hydrogel actuators improves from approximately 47 minutes without PEG to approximately 23 minutes with PEG, and further to 13 minutes with both PEG and a thinner active layer, a roughly 2x improvement with each step. These results make the response time on par with an at-home COVID test, while further refinement should reduce it substantially further.

In Supplementary Figures 14c and 14d, we present an alternative method of signal transduction that could provide even faster, sub-minute measurements. Supplementary Figure 14c shows the standard sensor response to various pH levels, with a T_{90} on the order of 15 minutes before the signal stabilizes. Supplementary Figure 14d shows the same data but zoomed into the first 30 seconds. Taking either ΔB_z after 30s or the initial slope of the response to be the sensor signal will still allow the user to discriminate between pH levels, even with additional noise introduced from the hydrogel settling after the solution change, as shown in Supplementary Fig. 14d. However, more characterization is necessary to determine the reproducibility, sensitivity, and dynamic range of these alternate, faster measurement methods.

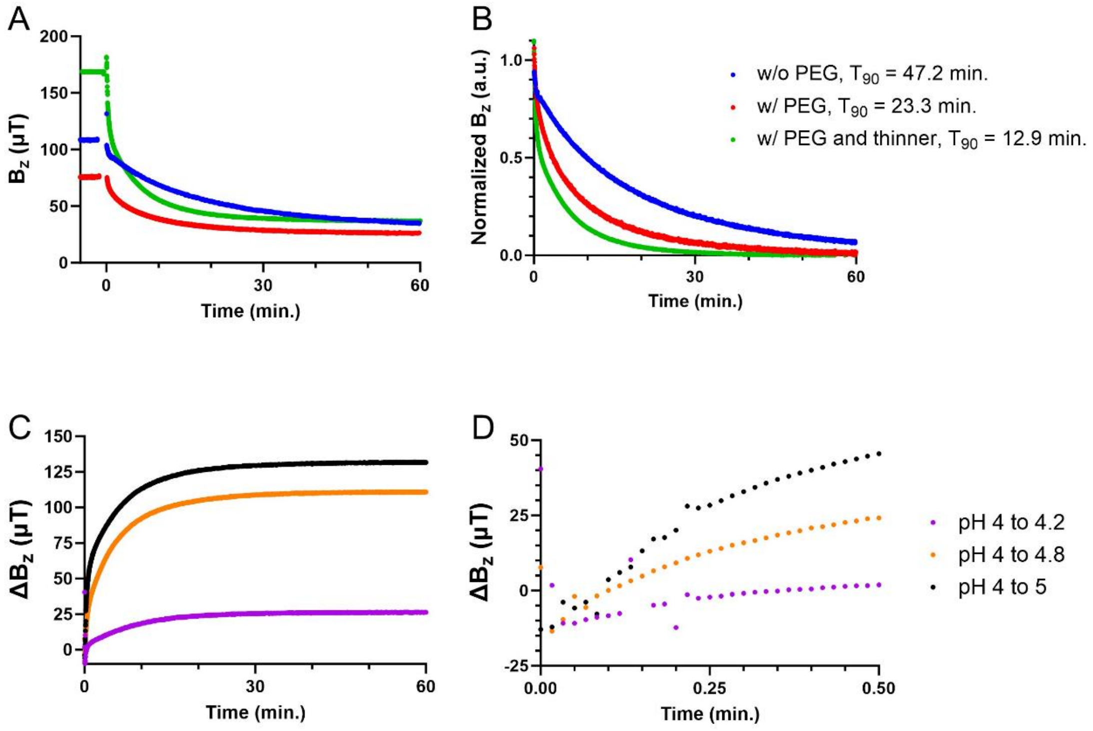

Supplementary Figure 14: Improving the hydrogel actuator response time. *(a)* The raw magnetometer reading (B_z) over time as the test solution is switched from pH 4 to pH 5 at time = 0 minutes. *(b)* Normalized response curves showing a clear improvement in the response time with the addition of PEG and with a thinner active layer. *(c)* ΔB_z over time when changing the solution to pH = 4.2, 4.8, or 5 from a baseline in pH 4 at time = 0 minutes. *(d)* ΔB_z over time showing a clear difference in the signal between pH 4.2, 4.8, and 5 in the first 30 seconds of response. Source data are provided as a Source Data file.

3. Supplementary Notes: detection limit results

To probe the detection limit of the smartphone magnetometer-based sensor, ΔB_z was recorded in response to five different concentrations of glucose, with 3 replicates each. For clarity, only three are shown in the main paper. Figure 3A is reproduced with error bars showing the range of ΔB_z recorded over the three replicates in Supplementary Fig. 15a. Supplementary Figure 15b shows the average ΔB_z for all five glucose concentrations recorded. The average ΔB_z was calculated at the end of each run, between 119 and 120 minutes for each concentration to determine a calibration curve, shown in Supplementary Fig. 15c. A linear regression was applied to determine the response equation. An estimate of the limit of detection (LOD) is then determined at approximately 8 mM based on the formula LOD = 3.3 σ/S, where S is the slope of the fit equation (0.039) and σ is the standard error of the Y-intercept determined from the linear regression (0.092). While this single-digit micromolar limit of detection is reached for the current proof-of-concept, future optimization of the design should decrease this value substantially.

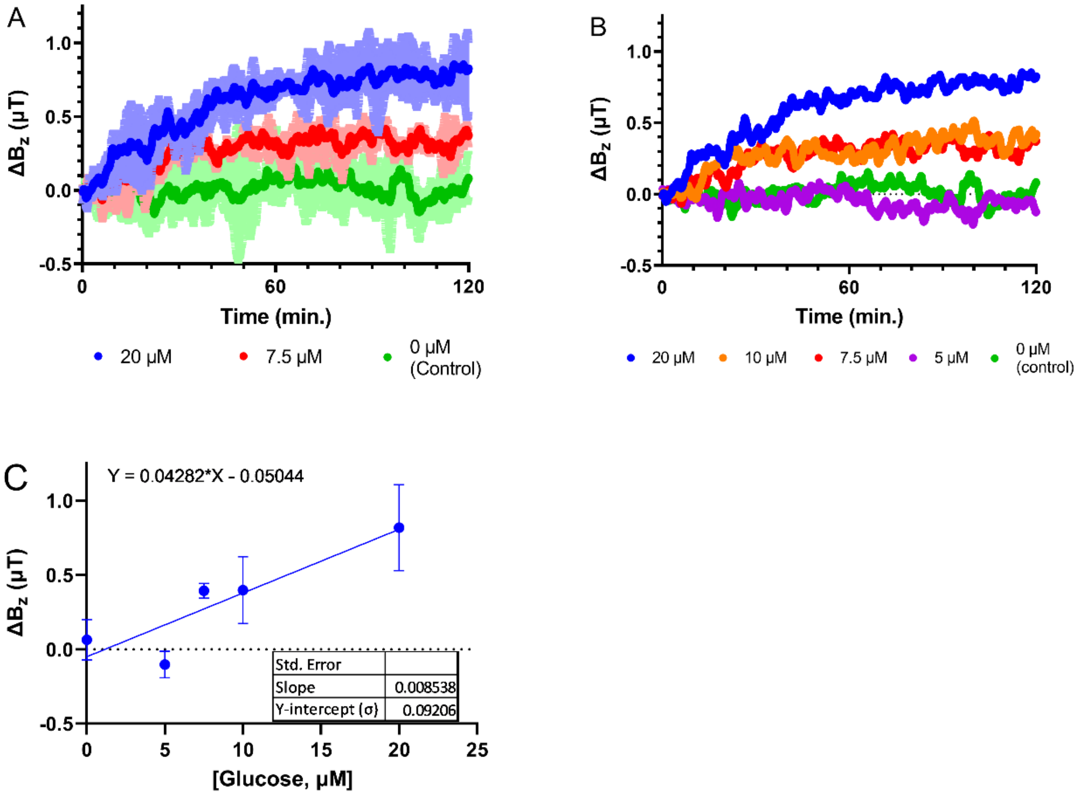

Supplementary Figure 15: Supplementary data for the detection limit test (Fig. 4a) in the main paper. *(a)* The average ΔB_z over time in response to different glucose concentration levels (labeled), and with error bars showing the range of ΔB_z recorded over three replicates for each concentration. *(b)* Full experimental data collected for detection limit testing, showing the average ΔB_z over time in response to five concentrations of glucose. *(c)* Calibration curve showing the average ΔB_z between 119 and 120 minutes in Supplementary Fig. 15b for the five glucose concentrations tested and fit with a linear trend. Source data are provided as a Source Data file.

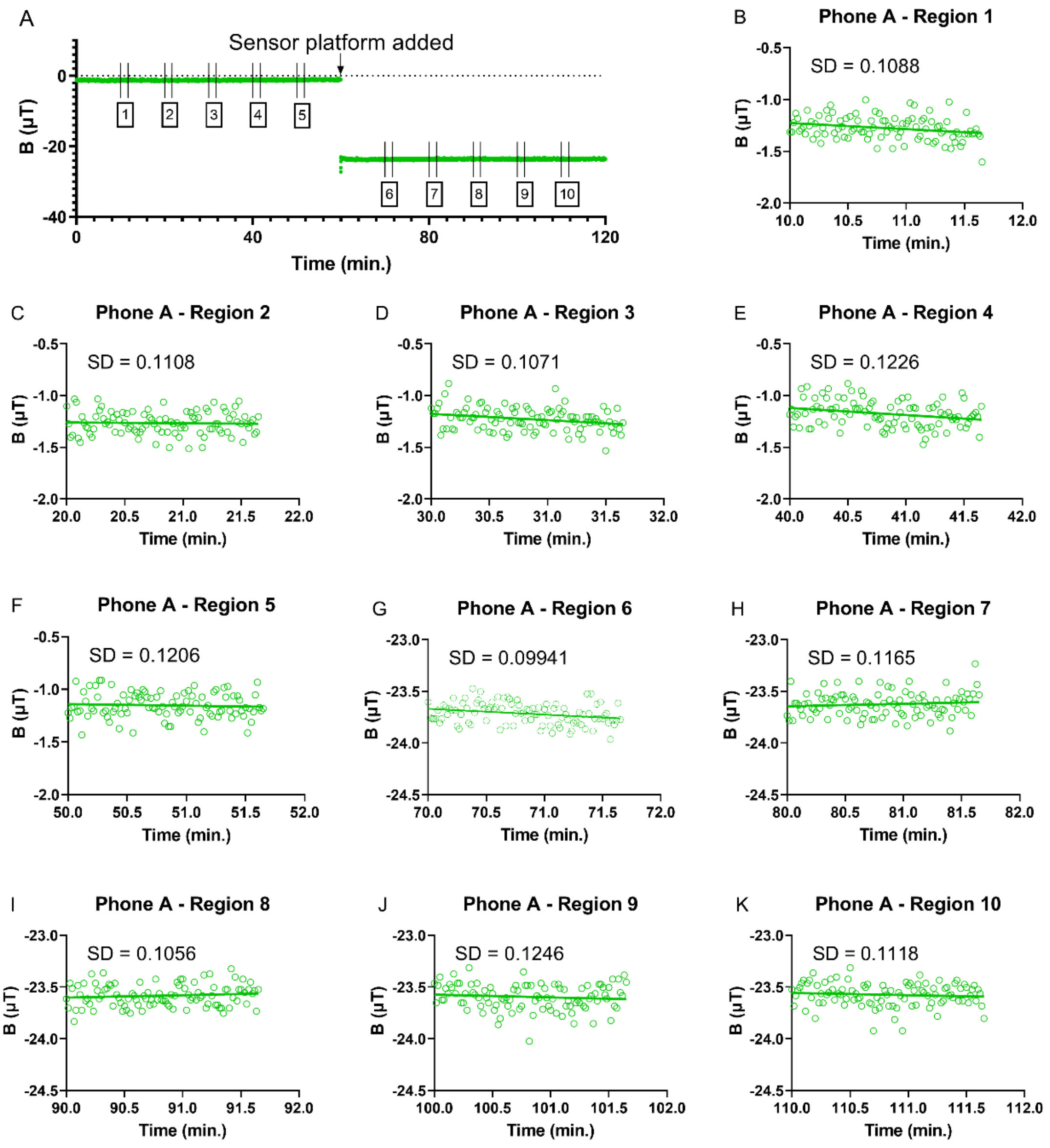

Supplementary Figure 16: Supplementary data for the noise analysis in Fig. 4b. On three different smartphones of the same model, the magnetometer was recorded for one hour without any sensor platform attached followed by one hour with the sensor platform attachment, with sample data from phone A shown here. *(a)* The z-direction field strength (B_z) as recorded by the magnetometer over the two-hour window, showing ten different 100-second-long regions where the noise of the system is interrogated, five before and five after the sensor platform attachment is added. *(b-k)* A linear trend is first fit to the data in each individual region to eliminate to first order any potential long-term background drift and then the noise level (standard deviation of the residuals, SD) is determined based on the formula $\mathrm{SD} = \sqrt{\sum \frac{\text{residual}^2}{n}}$, where *n* is the number of data points. Source data are provided as a Source Data file.

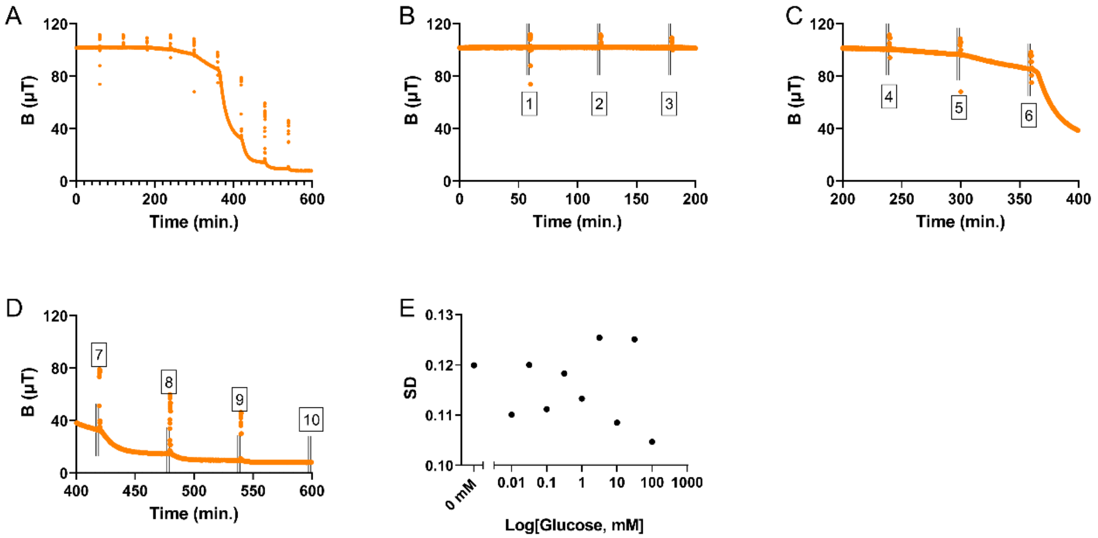

Supplementary Figure 17: Evaluation of noise from the dose/response data in Fig. 2b. *(a)* Raw magnetometer data from the sensor phone as the test solution is cycled through 0 mM, 10 mM, 31.6 mM, 100 mM, 316 mM, 1 mM, 3.16 mM, 10 mM, 31.6 mM, and 100 mM glucose for one hour each. *(b-d)* Raw magnetometer data from the sensor phone with regions labeled for noise analysis. The regions are 100 seconds long and start 57 minutes after each solution change so that the sensor is closest to equilibrium with the test solution equilibrated. *(e)* The noise for the region corresponding to each glucose concentration, where SD represents the noise level (standard deviation of the residuals) and is determined by the process shown in Supplementary 16. Since the $Nd_2Fe_{14}B$ particles move further away from the magnetometer with increasing glucose concentration, this data suggests that the noise level is not dependent on proximity of the $Nd_2Fe_{14}B$ particles to the magnetometer and, hence, is unlikely due to any random fluctuations in those particles' field or in their spatial location and/or orientation as might occur due to vibration or other mechanical disturbances. The data also suggest that the noise is independent of the state of curl of the hydrogel actuator. Source data are provided as a Source Data file.

4. Supplementary Discussion

4.1 Magnetic field as a function of bilayer curvature

As noted in the main manuscript, for small deflections of the hydrogel bilayer, the embedded disk of magnetized particles translates essentially vertically above the phone magnetometer and the field measured at magnetometer scales linearly with the hydrogel fractional expansion (see main paper Equation 3). In this limit of small hydrogel deflection, the sensor is both most linear and at its highest sensitivity, and if potential ranges of analyte concentrations are known, the hydrogel bilayer system should ideally be designed to operate within this regime.

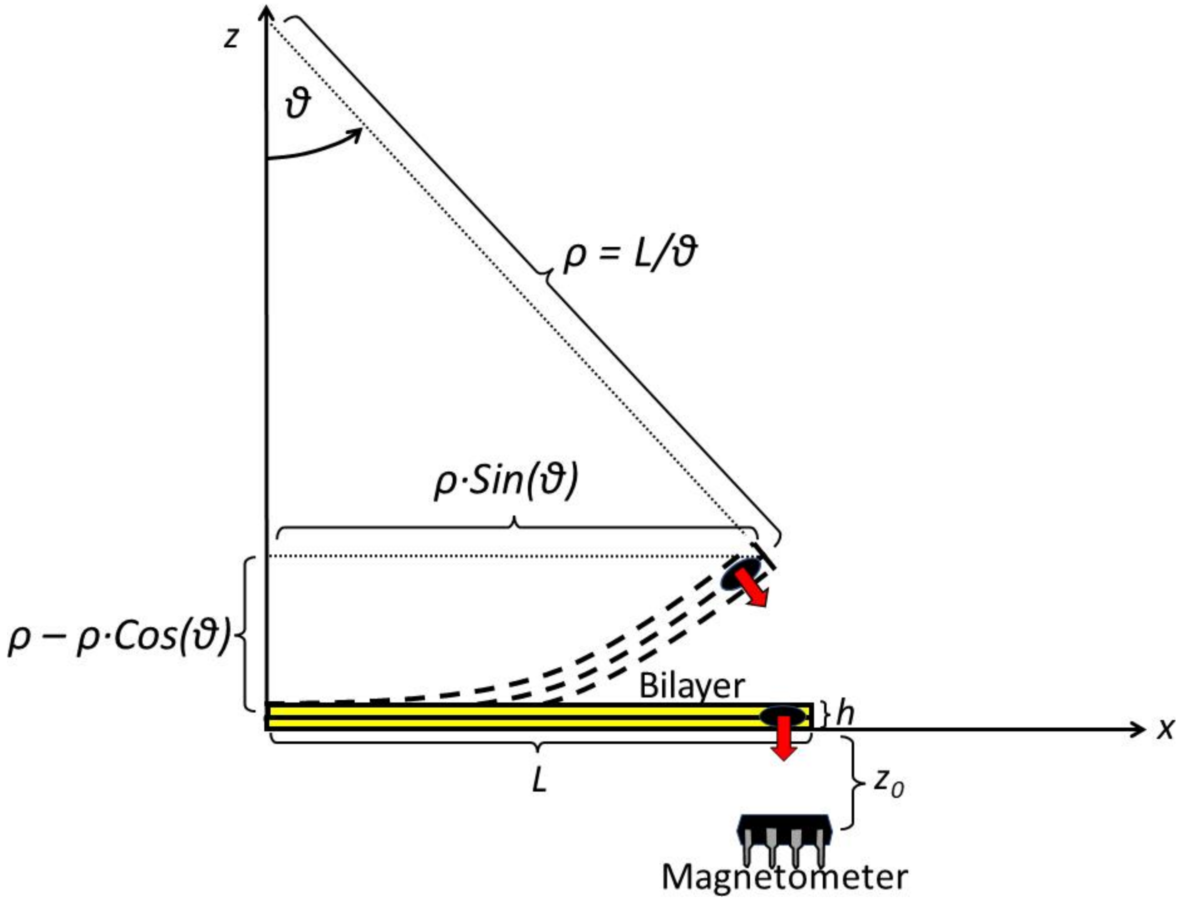

Supplementary Figure 18: Bilayer geometry. A flat (yellow), and subsequently curved bilayer of length *L* and thickness *h*, is shown sketched in the coordinate system used for all mathematical calculations in the paper. The end of the bilayer at the origin of the coordinate system is assumed fixed; the other end is free. The magnetization vector of the magnetized particles (black disk) at the free end of the bilayer strip is shown in red. A magnetometer chip buried below the end of the bilayer strip at depth Z_0 within a cellphone (whose top surface lies at $z = 0$) is also shown. For clarity, the bending angle is exaggerated from what would apply during normal sensor operation.

However, larger hydrogel bilayer deflections are also possible (as seen, for example, in Supplementary Movie 1, in Figs. 1e-1i and Supplementary Fig. 18). Larger deflections extend the dynamic range of the sensor but also lead to angular reorientations of the disk of magnetized particles, changing the direction of its magnetization with respect to the phone. Such deflections also cause not only vertical, but also

horizontal, translations of the disk. For these larger deflections a calibration look-up table can be used to translate between field measurements and analyte concentrations, but it is also possible to analytically predict the field dependences even in these more complex situations, which for completeness we derive below.

The bending of thin bilayer beams due to differences in the expansion / shrinking of their two composite layers was first studied by Timoshenko [34] for the case of a bimetallic strip that bends when heated. Here we translate that work to the case of a hydrogel bilayer strip, clamped at one end and with magnetized particles embedded at the opposite free end, with the goal of quantifying how hydrogel expansions / contractions in response to targeted analytes change the magnetic field measured by a magnetometer located beneath the free end of the bilayer strip. We start by rewriting the general expression found in [34] for the bilayer curvature, $1/r$, by replacing a difference in metallic thermal expansion factors with instead the fractional expansion, ε, of the active hydrogel layer in our bilayer strip to give:

$$\frac{1}{\rho} = \frac{6 \cdot (1 + h_{12})^2}{3(1 + h_{12})^2 + (1 + h_{12}E_{12})\left({h_{12}}^2 + 1/h_{12}E_{12}\right)} \cdot \frac{\varepsilon}{h} \tag{S1}$$

where h_{12} is the ratio of the top layer thickness h_1 to the bottom layer thickness h_2 in the bilayer strip, $h = h_1 + h_2$ is the total bilayer thickness, and E_{12} is similarly the ratio of Young's moduli of the top and bottom layer materials (see Supplementary Fig. 18). For concreteness, the above assumes that only the active layer dilates, but if both layers dilate simultaneously the same analysis still applies, only with ε replaced by the difference in expansion factors for the two hydrogel layers. It is also assumed that the hydrogel bilayer is oriented such that curling draws the magnetized end up away from the phone, rather than pushing down into the phone.

For simplicity, we begin with a bilayer comprising two layers of equal thickness. This will not always be true, in which case the full equation above can be returned to, but starting with equal layer thicknesses significantly simplifies the above:

$$\frac{1}{\rho} = \frac{3}{2} \cdot \frac{\varepsilon}{h} \tag{S2}$$

Here we have also assumed equal Young's moduli for the two layers. Again, this is unlikely to be true but, as can be seen by substitution, even large differences in layer thicknesses or Young's moduli yield only small changes to the 3/2 prefactor.

Assuming homogeneous hydrogel material composition within each layer, and hence uniform hydrogel expansion along the length of the layers, the bilayer will always take the form of an arc of a circle with radius ρ given above. (This remains true even for flat layers with $\varepsilon = 0$, where the circle has effectively infinite radius). As ε increases with increasing analyte concentration, the curvature increases and the hydrogel strip conforms to an increasing tighter arc of an increasing smaller circle. The opening angle θ (in radians) of this arc formed by a bilayer of length L is then:

$$\theta = \frac{L}{\rho} = \frac{3}{2} \cdot \frac{\varepsilon L}{h} \tag{S3}$$

Examples of the predicted bilayer curves are shown in Supplementary Fig. 19 for various ε and overlaid for comparison on real world examples copied from main paper Fig. 1e-1i.

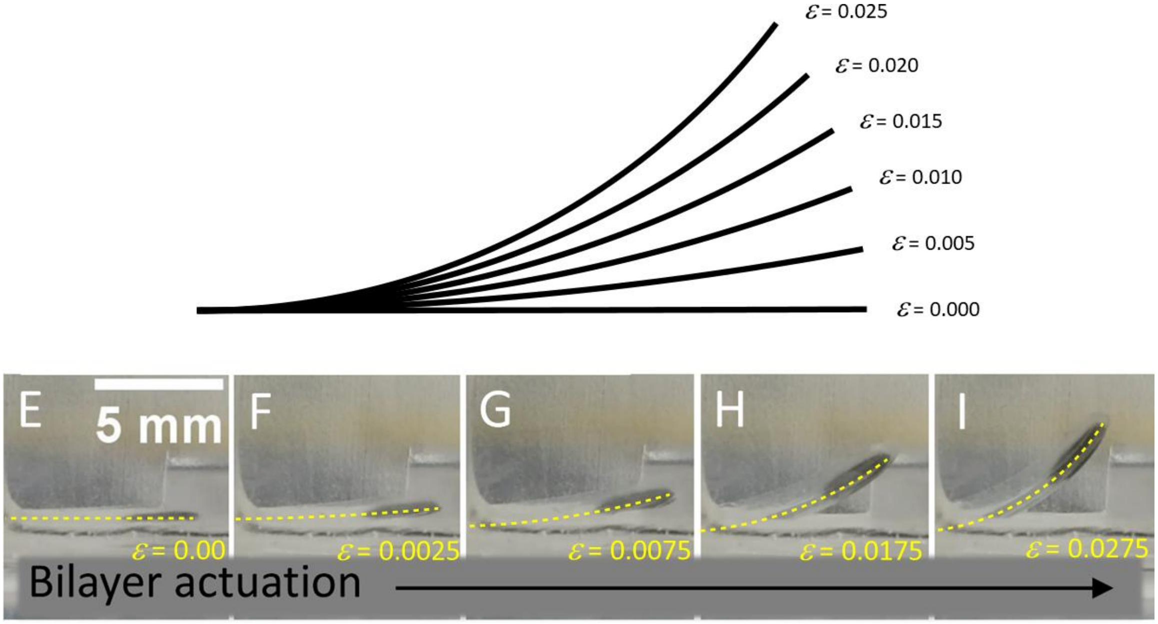

Supplementary Figure 19: Bilayer curves. Top shows predicted curve taken by a bilayer when one layer of that bilayer changes its length from L to $L(1+\varepsilon)$. Curves shown here are for a bilayer with L = 16 mm and h = 0.65 mm. Bottom overlays predicted bilayer curves onto still-frame images taken from movie S1 of actual bilayer curling (also shown in main paper Fig. 1e-1i). As discussed in the text, most of the sensor's useful range already occurs at small angles, but larger bilayer bendings are also included here for completeness.

Using the coordinate system sketched in Supplementary Fig. 18 with the clamped end of the bilayer taken to be at the origin, the vector position of the magnetized particles at the free end of the strip becomes, quite generally,

$$\{x, y, z\} = \rho \cdot \{\sin\theta, 0, 1-\cos\theta\} = L \cdot \left\{\frac{\sin\theta}{\theta}, 0, \frac{1-\cos\theta}{\theta}\right\} \qquad \text{(S4)}$$

while the vector magnetization, $\boldsymbol{M}$, of the disk, which has magnitude M and is magnetized normal to its surface along its axis of rotation, points in the direction:

$$\overrightarrow{\boldsymbol{M}} = M \cdot \{\sin\theta, 0, -\cos\theta\} \qquad \text{(S5)}$$

Meanwhile, in the sketched coordinate system (see Supplementary Fig. 18) with the surface of the phone at z=0, the phone magnetometer, embedded a depth z_0 into the phone, lies at position $\{L, 0, -Z_0\}$. The position of the magnetometer relative to the magnetized particles at the free end of the bilayer strip, can thus be described by a displacement vector:

$$\vec{\boldsymbol{r}} = L \cdot \left\{1 - \frac{\sin\theta}{\theta}, 0, \frac{(\cos\theta - 1)}{\theta} - \frac{Z_0}{L}\right\} \qquad \text{(S6)}$$

which points from the magnetized particles towards the magnetometer and has length equal to the distance between the particles and the magnetometer.

For small deflections, θ remains small and substituting the Taylor expansions sin $\theta \approx \theta$ and cos $\theta \approx 1 - \theta^2/2$ into the above yields $\boldsymbol{M} = M\cdot\{\theta, 0, -1\}$ and $\boldsymbol{r} = \{0, 0, -L\cdot\theta/2 - Z_0\}$. That is, the magnetization continues to point primarily straight downwards towards the magnetometer and there is essentially zero motion in *x* and *y* with the magnetic particles being displaced primarily in the *z*-direction away from the magnetometer by a distance of $L\cdot\theta/2$. Taking $\theta = 3\ \varepsilon\cdot L/2h$ from above, the displacement thus scales not simply proportionally to $\varepsilon\cdot L$, as would be true for a single homogeneous strip of hydrogel but instead proportionally to $3\varepsilon\cdot L^2/4h$, which constitutes a key advantage in using a bilayer hydrogel strip to enhance measurement sensitivity.

For these small deflections, which proceed primarily vertically and without any significant reorientation of the magnetization, the field from the disk-shaped collection of magnetized particles takes a simple analytic form, given by Equation 1 in the main paper. For larger motions of the hydrogel bilayer, however, the magnetometer no longer continues to lie along the axis of the disk; nor does the disk magnetization ***M*** point directly towards the magnetometer, invalidating the use of the simple on-axis circular current loop model used. At these larger distances, however, the field from the disk of particles can be well approximated with a simpler, spherical collection of particles, whose vector magnetic field ***B*** at position ***r*** relative to the center of that sphere is equivalent to that from a point magnetic dipole and given by:

$$\vec{\boldsymbol{B}}_{\text{dipole}}(\vec{\boldsymbol{r}}) = \frac{\mu_0}{4\pi}\left[\frac{3\hat{\boldsymbol{r}}(\hat{\boldsymbol{r}}\cdot\vec{\boldsymbol{m}}) - \vec{\boldsymbol{m}}}{|\vec{\boldsymbol{r}}|^3}\right] \tag{S7}$$

where μ_0 is the vacuum permeability and *m* is the total magnetic moment, and $\hat{\boldsymbol{r}}$ is the unit displacement vector. Or, rewritten in terms of an actual sphere of finite radius R_{sphere} with magnetization per unit volume, $M = m/\left(\frac{4}{3}\pi R_{sphere}^3\right)$:

$$\vec{\boldsymbol{B}}_{\text{dipole}}(\vec{\boldsymbol{r}}) = \frac{\mu_0 R_{sphere}^3}{3}\left[\frac{3\hat{\boldsymbol{r}}\left(\hat{\boldsymbol{r}}\cdot\vec{\boldsymbol{M}}\right) - \vec{\boldsymbol{M}}}{|\vec{\boldsymbol{r}}|^3}\right] \tag{S8}$$

Substituting for the vectors ***M*** and ***r*** from Equations S5 and S6, then yields general expressions for all three vector components of the magnetic field at the location of the magnetometer that remain valid for arbitrarily large curlings of the hydrogel bilayer:

$$B_x(\theta) = \frac{\mu_0 M R_{sphere}^3}{3L^3}\cdot\frac{\left[2\sin\theta\cdot\left(1-\frac{\sin\theta}{\theta}\right)^2 - 3\cos\theta\cdot\left(1-\frac{\sin\theta}{\theta}\right)\left(\frac{\cos\theta-1}{\theta}-\frac{Z_0}{L}\right) - \sin\theta\cdot\left(\frac{\cos\theta-1}{\theta}-\frac{Z_0}{L}\right)^2\right]}{\left[\left(1-\frac{\sin\theta}{\theta}\right)^2 + \left(\frac{\cos\theta-1}{\theta}-\frac{Z_0}{L}\right)^2\right]^{5/2}}$$

$$B_y(\theta) = 0 \tag{S9}$$

$$B_z(\theta) = \frac{\mu_0 M R_{sphere}^3}{3L^3}\cdot\frac{\left[\cos\theta\cdot\left(1-\frac{\sin\theta}{\theta}\right)^2 + 3\sin\theta\cdot\left(1-\frac{\sin\theta}{\theta}\right)\left(\frac{\cos\theta-1}{\theta}-\frac{Z_0}{L}\right) - 2\cos\theta\cdot\left(\frac{\cos\theta-1}{\theta}-\frac{Z_0}{L}\right)^2\right]}{\left[\left(1-\frac{\sin\theta}{\theta}\right)^2 + \left(\frac{\cos\theta-1}{\theta}-\frac{Z_0}{L}\right)^2\right]^{5/2}}$$

The measured field at the magnetometer can then be determined in terms of the fractional hydrogel expansion ε, which itself depends on the analyte concentration being tested, by substituting either the simple expression $\theta = \varepsilon\cdot(3L/2h)$, or by starting from the more complete expression in Equation S1, as appropriate.

While the above is valid for any θ, it is instructive to compare its behavior for small θ with that for the disk-shaped magnet given in the main paper. Taylor expanding the above for small θ gives:

$$B_x(\theta) \approx -\frac{\mu_0 M R_{sphere}^3}{3} \cdot \frac{\theta}{Z_0^3}$$

$$B_y(\theta) = 0 \tag{S10}$$

$$B_z(\theta) \approx \frac{\mu_0 M R_{sphere}^3}{3} \cdot \left(-\frac{2}{Z_0^3} + \frac{3\theta \cdot L}{Z_0^4}\right)$$

As detailed in the main paper, for small displacements Δz, the change in field is $\Delta B = (\partial B/\partial z) \cdot \Delta z$ which now in terms of σ becomes $\Delta B = (\partial B/\partial \theta) \cdot (\partial \theta/\partial z) \cdot \Delta z$. As shown above, for small θ the z-position of the magnet is described by $z \approx L \cdot \theta/2$, giving $\partial \theta/\partial z = 2/L$. Taking $\Delta z = 3\varepsilon L^2/4h$, then yields:

$$\Delta B_x \approx -\mu_0 M R_{sphere}^3 \cdot \frac{L}{2hZ_0^3} \cdot \varepsilon$$

$$\Delta B_y = 0 \tag{S11}$$

$$\Delta B_z \approx \mu_0 M R_{sphere}^3 \cdot \frac{3L^2}{2hZ_0^4} \cdot \varepsilon$$

To facilitate better comparison with disk-shaped magnets, the total magnetic moments of sphere and disk can be matched by equating their volumes via $R_{sphere}^3 = 3R_{disk}^2 d/4$, where d was the disk thickness. Note that in the limit $R_{disk} \ll Z_0$, where from the vantage point of the magnetometer the disk arrangement of magnetic particles becomes indistinguishable from a spherical one, ΔB_z becomes identically equivalent to that of Equation 2 in the main paper, as expected. Alternatively, setting $R_{disk} = \sqrt{2/3} \cdot Z_0$ (as detailed in the main paper), and substituting into the above yields:

$$\Delta B_x \approx -\frac{\mu_0 M d L}{4hZ_0} \cdot \varepsilon$$

$$\Delta B_y = 0 \tag{S12}$$

$$\Delta B_z \approx \frac{3\mu_0 M d L^2}{4hZ_0^2} \cdot \varepsilon$$

As expected, this has the same form as Equation 3 in the main paper, only with a different numerical prefactor owing to the spherical, rather than disk-shaped, magnetic particle distribution. Interestingly, while the current work used a disk-shaped magnetic particle ensemble (because it was simplest to fabricate), comparing the above with Equation 3 shows that arranging the same quantity of magnetic particles into a sphere, rather than a disk, could in the future enhance sensor sensitivity a further 3-fold.

4.2 Range of validity of on-axis disk field model (main paper Equation 1)

While the above gives general field expressions for arbitrary bilayer curling, the simpler on-axis disk field model presented in the main paper can be applied over a large portion of the sensor's dynamic range, as shown here.

Equation 1 in the main paper, reproduced here for the field at a distance $z + Z_0$ away from the center of a magnetized disk of magnetization *M*, radius *R*, and thickness *d*, is given by:

$$B_z(z) = -\frac{\mu_0 M d}{2} \cdot \frac{R^2}{((z + Z_0)^2 + R^2)^{\frac{3}{2}}} \tag{S13}$$

Substituting in the optimal disk radius defined in the paper, $R = \sqrt{2/3} \cdot Z_0$, gives:

$$B_z(z) = -\frac{\mu_0 M d z_0^2}{3\left((z + Z_0)^2 + \frac{2}{3} Z_0^2\right)^{3/2}} \tag{S14}$$

which represents a field that rapidly decays in space. Basic rearrangement shows that $B_z(z)$ falls to $1/n$ of its initial value, $B_z(z=0)$, when $z = \left(\sqrt{(5n^{2/3} - 2)/3} - 1\right) \cdot Z_0$. For example, $B_z(z)$ falls to one half of its initial value at a distance of $z = 0.4Z_0$ above the surface of the phone. That is, for a magnetometer embedded at a depth z_0 of around 3 to 5 mm beneath the surface of the phone, the field can be made to drop by 50% by raising the disk magnet's *z*-position just 1 to 2 mm above the phone surface. Assuming even a relatively short hydrogel bilayer strip with *L* = 20 mm, this corresponds to a change in orientation angle of the plane of the disk of order 5 to 10 degrees from horizontal and a shift in the magnet's x-position of no more than 0.1 mm, as can both be determined through use of Equation S4. At 0.1 mm, this lateral shift is both far smaller than the size of the magnetic disk and negligible compared to the disk's *z*-translation. Meanwhile, the 5-to-10-degree reorientation of the magnetization direction yields less than a 2 percent reduction in the B_z field component, which scales with the cosine of the reorientation angle. That is, over at least half of the sensor's dynamic range, the simple model of a disk translating purely vertically without any reorientation, as given in Equation 1, remains a good approximation.

Equivalent analysis shows that even a 75% field reduction still leads to a mostly negligible 0.5 mm lateral shift in position, albeit this time of order 20-degree reorientation corresponding to or order 10% decrease in the B_z field component. While still not a large error now over even a full ¾ of the sensor dynamic range, good approximation can still be achieved with the horizontally oriented on-axis disk model of Equation 1 by adding a $\cos\theta$ correction factor to better account for the (slightly) changed B_z field projection. Recasting Equation 1 then in terms of θ, recalling that $z = (1 - \cos\theta) \cdot (L/\theta)$ from Equation S4, gives the more complex, but more accurate approximation:

$$B_z(\theta) = -\frac{\mu_0 M d Z_0^2}{3\left(\left(\frac{1 - \cos\theta}{\theta} \cdot L + Z_0\right)^2 + \frac{2}{3} Z_0^2\right)^{3/2}} \cdot \cos\theta \tag{S15}$$

Or, substituting $\theta = 3\varepsilon L/2h$ from Equation S3 then yields the measured B_z field at the magnetometer directly in terms of the hydrogel fractional expansion, ε:

$$B_z(\varepsilon) = -\frac{\mu_0 M d Z_0^2}{3\left(\left(\frac{1 - \cos(3\varepsilon L/2h)}{3\varepsilon} \cdot 2h + Z_0\right)^2 + \frac{2}{3} Z_0^2\right)^{3/2}} \cdot \cos\frac{3\varepsilon L}{2h} \tag{S16}$$

which applies over the majority of the sensor's useful dynamic range. To better understand this equation, the predicted B_z field at the location of a magnetometer embedded a distance z_0 = 5 mm below the phone surface is shown plotted in Supplementary Fig. 20 as a function of the hydrogel expansion ε for a typical hydrogel bilayer of length L = 20 mm and thickness h = 0.5 mm. Focusing on the sensor's initial, most sensitive, measurement range, an overlaid straight line fit on the actual field shows that the sensor response deviates little from linearity over the first 50% of its dynamic range (see figure inset).

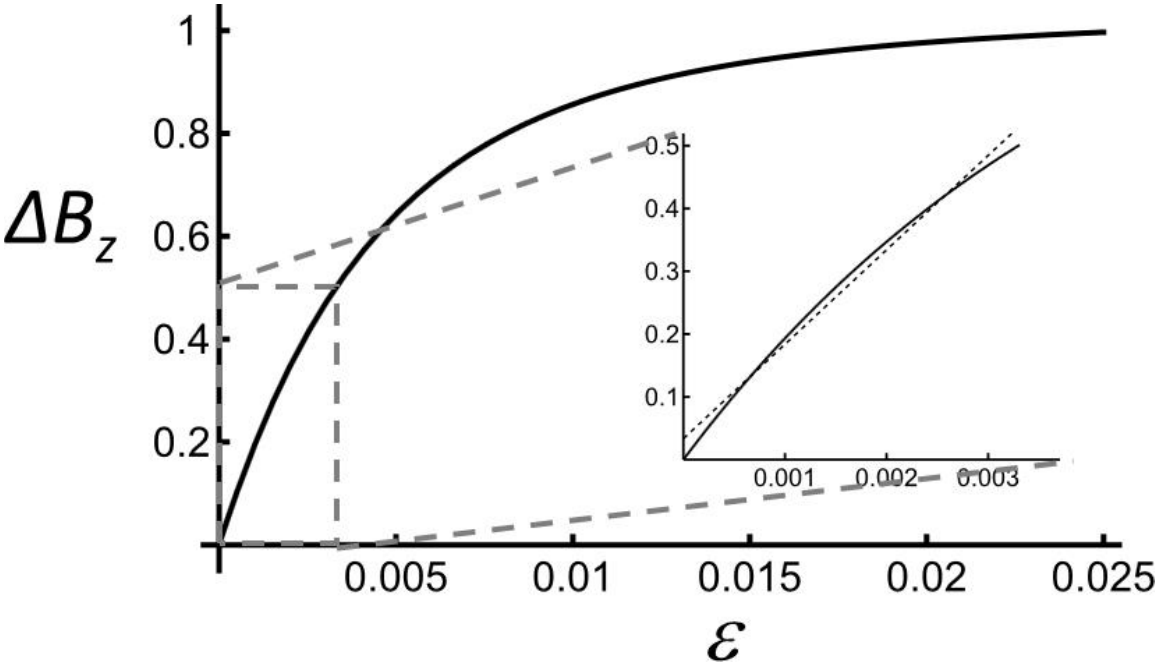

Supplementary Figure 20: Field change ΔB_z as a function of hydrogel fractional expansion ε. Inset overlays a straight line fit, showing degree of linearity over first 50% of sensor dynamic range. Plot is based on equation S16 and shown for a bilayer with L = 20 mm and h = 0.5 mm.

4.3 Comparison of bilayer vs monolayer

Comparing the above to that which would result from a monolayer gel, helps quantify the advantage of a bilayer hydrogel geometry. We perform this analysis here for monolayers oriented both horizontally (like the bilayer) and vertically above the phone surface, as shown schematically in Supplementary Fig. 21.

i) Horizontally oriented monolayer

If the above bilayer is replaced by a simple (monolayer) hydrogel, positioned identically to the bilayer above the phone and with a length that scales with the same fractional hydrogel expansion ε as the active layer in the above bilayer, then Equation S4 above can be rewritten for the position of the magnetized particles in a single layer system as:

$$\{x, y, z\} = \{L(1+\varepsilon), 0\,, 0\} \qquad \text{(S17)}$$

with the position of the magnetometer relative to the magnetized particles now being described by the displacement vector:

$$\vec{\boldsymbol{r}} = \{-\varepsilon L, 0, -Z_0\} \qquad \text{(S18)}$$

Since expansion/contraction of a single homogeneous layer involves no change in curvature or angular orientation, Equation S5 for the vector magnetization, ***M***, of the disk, simplifies to:

$$\vec{\boldsymbol{M}} = M \cdot \{0, 0, -1\} \qquad \text{(S19)}$$

Substituting into Equation S8, then yields general expressions for the magnetic field at the location of the magnetometer, this time due to an expanding single hydrogel layer:

$$B_x(\varepsilon) = -\frac{\mu_0 M R_{sphere}^3}{L^4} \cdot \frac{\varepsilon Z_0}{\left[\varepsilon^2 + \left(\frac{Z_0}{L}\right)^2\right]^{5/2}}$$

$$B_y(\varepsilon) = 0 \qquad \text{(S20)}$$

$$B_z(\varepsilon) = \frac{\mu_0 M R_{sphere}^3}{3L^3} \cdot \frac{\varepsilon^2 - 2\left(\frac{Z_0}{L}\right)^2}{\left[\varepsilon^2 + \left(\frac{Z_0}{L}\right)^2\right]^{5/2}}$$

which for small ε become:

$$B_x(\varepsilon) = -\frac{\mu_0 M R_{sphere}^3}{z_0^4} \cdot L \cdot \varepsilon$$

$$B_y(\varepsilon) = 0 \qquad \text{(S21)}$$

$$B_z(\varepsilon) = -\frac{\mu_0 M R_{sphere}^3}{3Z_0^3} \cdot \left(2 - 6\left(\frac{L}{Z_0}\right)^2 \cdot \varepsilon^2\right)$$

For small displacements, the change in field is $\Delta B = (\partial B/\partial x) \cdot \Delta x$ which in terms of ε becomes $\Delta B = (\partial B/\partial \varepsilon) \cdot (\partial \varepsilon/\partial x) \cdot \Delta x$, where we have now switched from a motion that was primarily in the *z*-direction for a bilayer expansion to one that now proceeds in the *x*-direction for a single layer, simply expanding hydrogel. Substituting $\partial \varepsilon/\partial x = 1/L$ and $\Delta x = \varepsilon L$, then yields:

$$\Delta B_x \approx -\mu_0 M R_{sphere}^3 \cdot \frac{L}{Z_0^4} \cdot \varepsilon$$
$$\Delta B_y = 0 \qquad (S22)$$
$$\Delta B_z \approx \mu_0 M R_{sphere}^3 \cdot \frac{4L^2}{Z_0^5} \cdot \varepsilon^2$$

Whereas the field change for a bilayer occurs primarily in B_z, for a monolayer oriented identically to a (initially flat) bilayer, the field change now occurs primarily in B_x. Comparing this field change to that of an equivalent bilayer system (see Equation S11) shows that for the same hydrogel expansion, the bilayer system yields a field change that is larger than that of an equivalently oriented and positioned monolayer system by a factor of $(3/2) \cdot (L/h)$. That is, for a typical bilayer with *L* = 20 mm and *h* = 0.5 mm, the bilayer yields nearly two orders of magnitude greater sensitivity, with this relative advantage increasing for longer and/or thinner bilayer strips.

ii) Vertically oriented monolayer

A monolayer system could also alternatively be oriented vertically, rather than laying horizontally, on the phone surface as analyzed above. In this case Equation S4 for the position of the magnetized particles becomes:

$$\{x, y, z\} = \{L, 0, L(1 + \varepsilon)\} \qquad (S23)$$

with the position of the magnetometer relative to the magnetized particles now being described by the displacement vector:

$$\vec{\boldsymbol{r}} = \{0, 0, -Z_0 - L(1 + \varepsilon)\} \qquad (S24)$$

Regardless of whether the monolayer is oriented horizontally or vertically, the vector magnetization, ***M***, of the disk remains:

$$\vec{\boldsymbol{M}} = M \cdot \{0, 0, -1\} \qquad (S25)$$

Substituting into Equation S8, then yields general expressions for the magnetic field at the location of the magnetometer, this time due to an expanding single hydrogel layer that is vertically oriented:

$$B_x(\varepsilon) = 0$$
$$B_y(\varepsilon) = 0 \qquad (S26)$$
$$B_z(\varepsilon) = -\frac{\mu_0 M R_{sphere}^3}{3} \cdot \frac{2}{[Z_0 + L(1 + \varepsilon)]^3}$$

which for small ε become:

$$B_x(\varepsilon) = 0$$

$$B_y(\varepsilon) = 0 \tag{S27}$$

$$B_z(\varepsilon) = -\frac{\mu_0 M R_{sphere}^3}{3(Z_0 + L)^3} \cdot \left(2 - \frac{6\varepsilon L}{Z_0 + L}\right)$$

For small displacements, $\Delta B = (\partial B/\partial z) \cdot \Delta z$, or in terms of ε, $\Delta B = (\partial B/\partial \varepsilon) \cdot (\partial \varepsilon/\partial z) \cdot \Delta x$. Substituting $\partial \varepsilon/\partial z = 1/L$ and $\Delta z = \varepsilon L$, then yields:

$$\Delta B_x = 0$$

$$\Delta B_y = 0 \tag{S28}$$

$$\Delta B_z \approx \mu_0 M R_{sphere}^3 \cdot \frac{2L}{(z_0 + L)^4} \cdot \varepsilon$$

Comparing this field change to that of an equivalent bilayer system (see Equation S11) shows that for the same hydrogel expansion, a bilayer system yields a field change that is larger than that of an equivalently sized, vertically oriented monolayer system by a factor of $(3/4) \cdot (1 + L/Z_0)^4 \cdot (L/h)$. For example, for the same typical bilayer with L = 20 mm and h = 0.5 mm, the bilayer yields over four orders of magnitude greater sensitivity. These results are summarized in Supplementary Fig. 21.

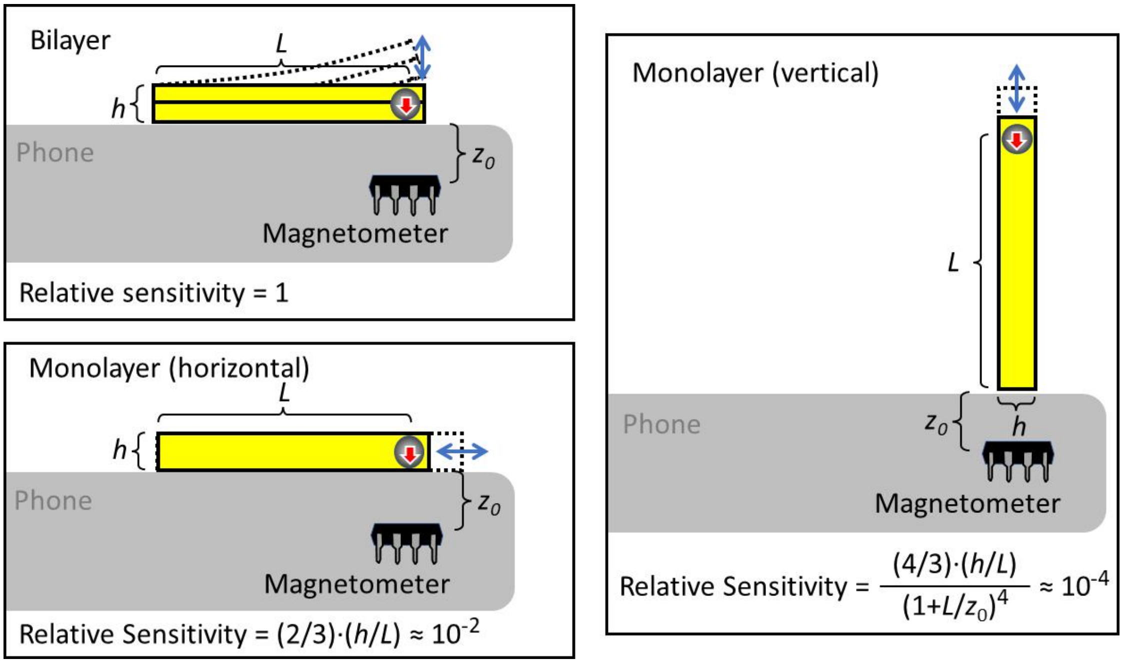

Supplementary Figure 21: Comparison of bilayer versus monolayer sensitivity. For equivalent hydrogel sizes and expansions, and equal magnetizations (red arrow), a bilayer moves the embedded magnetic materials (grey circles) further than a monolayer, resulting in larger field changes and higher sensitivity. Relative sensitivity scalings are shown comparing horizontal and vertical monolayers against a bilayer design. Numerical estimates are for a typical bilayer of size L = 20 mm, h = 0.5 mm and a magnetometer offset Z_0 = 5 mm. Relative sensitivity of the bilayer system increases further by increasing hydrogel length and/or reducing hydrogel thickness.

4. Supplementary Tables: Cost estimates

Supplementary Table 1: Cost estimate for glucose Hydrogel Actuator

**Cost per individual hydrogel actuator**		
**Component**	**Glucose Smart Hydrogel**	**Inert Hydrogel**
Acrylamide	0.06¢	0.28¢
BIS	0.01¢	0.03¢
DMA	0.04¢	0.08¢
3-APB	13.24¢	N/A
LAP	0.53¢	1.76¢
Total cost of above monomers	13.88¢	2.15¢
$Nd_2Fe_{14}B$	0.04¢	
Total:	$0.16	

Supplementary Table 2: Cost estimate for pH Hydrogel Actuator

**Cost per individual hydrogel actuator**		
**Component**	**pH Smart Hydrogel**	**Inert Hydrogel**
Acrylamide	1.13¢	0.21¢
Acrylic Acid	0.04¢	N/A
Poly(ethylene glycol) diacrylate, average molecular weight 700	1.07¢	0.20¢
2,2-Dimethoxy-2-phenylacetophenone	0.20¢	0.04¢
Total cost of above monomers	2.44¢	0.45¢
$Nd_2Fe_{14}B$	0.04¢	
Total:	$0.03	

5. Supplementary Methods

Synthesis of Glucose Hydrogel Actuators. A 'T' shaped poly-dimethylsiloxane (PDMS) well was first created and then used as a mold to form the glucose hydrogel actuators. The top, horizontal segment was 12 mm in width, 4 mm in height, and 0.5 mm in depth and the bottom, vertical segment was 10 mm in height, 4 mm in width, and 0.5 mm in depth. Corners were rounded to reduce any potential stress buildup in the hydrogel shape. A thin, 0.05 mm tall bar protruding from the bottom of the well was used to separate the horizontal segment from the vertical segment. 24 mL of inert hydrogel precursor was placed in the top, horizontal segment of the well and pinned to the walls of the well and the separating bar via naturally occurring capillary wetting forces. 20 mL of glucose-responsive precursor was spread on the vertical segment and similarly pinned to the walls and gently connected to the inert precursor in the horizontal segment. The bottom layer was then partially cured under UV light (365 nm, 4W) with N_2 gas flowing for 40 seconds. Then, 2 mg of silica-coated $Nd_2Fe_{14}B$ particles were mixed into 22 mL of inert precursor and deposited on top of an immobilized magnet, placed directly beneath the PDMS well, to magnetically attract the particles into a disc shape near the tip of the vertical arm, before spreading the liquid inert precursor to the walls of the well along the T-shape. Due to shrinkage of the bottom layer during curing, the same well walls were used to pin both the top and bottom layers. Both layers were then fully cured and bonded under UV light (365 nm, 4W) with N_2 gas flowing for 10 minutes.

Synthesis of pH Hydrogel Actuators. A similar 'T'-shaped PDMS well was used to form the pH hydrogel actuators. The top, horizontal segment was 16.5 mm in width, 5.5 mm in height, and 0.5 mm in depth and the bottom, vertical segment was 15 mm in height, 5.5 mm in width, and 0.5 mm in depth. Corners were rounded to reduce any potential stress buildup in the hydrogel shape. Due to a difference in degree of initial swelling when placed in solution after curing, pH hydrogel actuators were synthesized in larger wells to achieve similar sized structures to the glucose counterparts. 110 mL of inert hydrogel was placed in the well and pinned to all edges of the 'T' shape before partially curing under UV light (365 nm, 4W) with N_2 gas flowing for 20 seconds. Then, 2 mg of silica coated $Nd_2Fe_{14}B$ particles were mixed into 10 mL of pH hydrogel precursor and deposited on top of an immobilized magnet, placed directly beneath the PDMS well, to magnetically attract the particles into a disc shape near the tip of the vertical arm, before pinning the pH hydrogel precursor to the edges along the vertical segment of the well only. Both layers were then fully cured and bonded under UV light (365 nm, 4W) with N_2 gas flowing for 5 minutes.

Smartphone data collection. Two smartphones were typically used for data collection; one with the sensor platform attached (sensor phone) and a second smartphone that was placed near nearby and with no attachments to control for potential variations in background magnetic field (background phone). Magnetometer data was collected on both the sensor phone and background phone using Physics Toolbox Pro app for all experiments, with initial calibration to subtract off Earth's background field. All other apps, internet, Bluetooth, and other phone functions were turned off during testing to prevent CPU usage from affecting the magnetometer readings[1]. A background-subtracted B_t was then determined with the formula $B_t = \sqrt{{B_x}^2 + {B_y}^2 + {B_z}^2}$, where B_x, B_y, and B_z are the background-subtracted field readings (sensor phone reading minus control phone reading) in the x, y, and z directions. Although background subtraction was always performed, it was found empirically to almost never be needed, not making any significant difference to the data unless there was a significant external magnetic event during data collection (which happened only rarely and only when doing very long runs over many hours).

Detection Limit Testing. A single glucose hydrogel actuator was used to collect the data shown in Fig. 3A. For each experiment, the hydrogel actuator was given four hours to equilibrate in 5 mL of HEPES/NaOH buffer, pH 7.4, with 0 mM glucose. Then, a small amount of HEPES/NaOH buffer, pH 7.4, with 2 mM glucose was added to the test solution (12.5 mL, 18.8 mL, 25.1, or 50.4 mL) to raise the total glucose concentration to the desired levels (5 mM, 7.5 mM, 10 mM or 20 mM, respectively), followed by gently mixing the solution with a 2 mL transfer pipette to achieve a uniform

glucose concentration throughout. To control for the effects of the injecting and mixing process, 50.4 mL of HEPES/NaOH buffer, pH 7.4, with 0 mM glucose was added to the solution, followed by the same mixing procedure. Each glucose concentration and the control were tested in triplicate and in random order, and the average is shown in Fig. 4b. A 60 second moving average was applied to the data sets, and ΔB_z is calculated from a baseline of the field strength reading at 3.5 minutes to allow time for the actuator to settle after the injection and mixing process.

Supplementary References

1. Matyunin, N. *et al.* MagneticSpy: Exploiting magnetometer in mobile devices for website and application fingerprinting. *Proceedings of the ACM Conference on Computer and Communications Security* 135–149 (2019) doi:10.1145/3338498.3358650.
