# Supplementary material for: Quantitative, high-sensitivity measurement of liquid analytes using a smartphone compass
Source: Nat Commun. 2024 Mar 30;15:2801. doi: 10.1038/s41467-024-47073-2 (PMC10981709; doi:10.1038/s41467-024-47073-2)
Supplement: Supplementary file 3 — Description of Additional Supplementary Files [file 41467_2024_47073_MOESM3_ESM.docx]

**Supplementary Movie 1.** Timelapse video of glucose hydrogel actuator curling in response to 20 mM glucose.
